# Toxic Effect of Silica Nanoparticles on Endothelial Cells through DNA Damage Response via Chk1-Dependent G2/M Checkpoint

**DOI:** 10.1371/journal.pone.0062087

**Published:** 2013-04-19

**Authors:** Junchao Duan, Yongbo Yu, Yang Li, Yang Yu, Yanbo Li, Xianqing Zhou, Peili Huang, Zhiwei Sun

**Affiliations:** 1 School of Public Health, Capital Medical University, Beijing, P.R. China; 2 School of Public Health, Jilin University, Changchun, Jilin, P.R. China; Florida State University, United States of America

## Abstract

Silica nanoparticles have become promising carriers for drug delivery or gene therapy. Endothelial cells could be directly exposed to silica nanoparticles by intravenous administration. However, the underlying toxic effect mechanisms of silica nanoparticles on endothelial cells are still poorly understood. In order to clarify the cytotoxicity of endothelial cells induced by silica nanoparticles and its mechanisms, cellular morphology, cell viability and lactate dehydrogenase (LDH) release were observed in human umbilical vein endothelial cells (HUVECs) as assessing cytotoxicity, resulted in a dose- and time- dependent manner. Silica nanoparticles-induced reactive oxygen species (ROS) generation caused oxidative damage followed by the production of malondialdehyde (MDA) as well as the inhibition of superoxide dismutase (SOD) and glutathione peroxidase (GSH-Px). Both necrosis and apoptosis were increased significantly after 24 h exposure. The mitochondrial membrane potential (MMP) decreased obviously in a dose-dependent manner. The degree of DNA damage including the percentage of tail DNA, tail length and Olive tail moment (OTM) were markedly aggravated. Silica nanoparticles also induced G2/M arrest through the upregulation of Chk1 and the downregulation of Cdc25C, cyclin B1/Cdc2. In summary, our data indicated that the toxic effect mechanisms of silica nanoparticles on endothelial cells was through DNA damage response (DDR) via Chk1-dependent G2/M checkpoint signaling pathway, suggesting that exposure to silica nanoparticles could be a potential hazards for the development of cardiovascular diseases.

## Introduction

Silica nanoparticles have been found extensive applications in biomedical and biotechnological fields [1], such as medical diagnostics, drug delivery, gene therapy, biomolecules detection, photodynamic therapy and bioimaging [2], [3], [4]. This adds to the increasing industrial exposure to silica nanoparticles during production, transportation, storage, and consumer use by which human exposure and environmental burden were obviously increased. Epidemiological evidences link air pollution with fine particles in which silica is inorganic components to increase the morbidity and mortality of cardiovascular diseases [5], [6], [7]. In addition, several studies have shown translocation of ultrafine particles from the lungs to extrapulmonary organs via the systemic circulation [8], [9], [10]. Thus, endothelial cells could be directly exposed to ultrafine particles. Moreover, silica nanoparticles as carriers of drug delivery or gene therapy are generally injected into the body intravenously and directly contacted with endothelial cells. The single layer of endothelial cells that lines the lumen of all blood vessels is recognized to be not only a barrier between circulating blood and the vessel wall, but also a critical factor for the maintenance of vascular function and homeostasis [11]. Therefore, it is important to understand the interaction between silica nanoparticles and endothelial cells.

The human umbilical vein endothelial cells (HUVECs) line isolated from the umbilical cord by collagenase digestion has been used for in vitro studies of endothelial cells function [12]. Unfortunately, most previous studies focused on the cytotoxicity induced by silica nanoparticles using a wide range of different cells lines rather than endothelial cell line [13], [14], [15]. Although recently reports have shown that HUVECs exposure to silica nanoparticles could induce reactive oxygen species (ROS), inflammatory cytokines and von Willebrand factor (VWF) [16], [17], [18], information about the toxic effect and its mechanisms of silica nanoparticles on endothelial cells is still limited. Our previous study confirmed that silica nanoparticles caused oxidative DNA damage and cell cycle arrest in human hepatoma (HepG2) cells [19]. However, as far as we know, whether the silica nanoparticles could also induce endothelial cells toxic effect through oxidative DNA damage or cell cycle arrest has not been reported.

Mammalian cells are frequently at risk of DNA damage from a variety of endogenous and exogenous sources, including reactive oxygen species, ultraviolet light, background radiation and environmental factors [20]. To protect their genomes from this assault, cells have evolved complex mechanisms known as DNA damage response (DDR) that act to rectify damage and minimize the probability of lethal or permanent genetic damage [21]. DDR encompass multiple repair mechanisms and signal transduction pathways that effect cell cycle checkpoint arrest and/or apoptosis [22]. These regulatory mechanisms involving an intricate network of protein kinase signaling pathways are central to the maintenance of genomic integrity and basic viability of the cells [23]. Intact DDR pathways are very critical for preventing the replication of damaged DNA templates and transmission of mutations to daughter cells. Whereas defects in DDR will result in accumulation of genetic mutations, gene amplification, and chromosomal alterations, which in turn contribute to malignant transformation and tumorigenesis [24]. Therefore, it is necessary to clarify the basic molecular mechanism of silica nanoparticles-induced DDR pathways in endothelial cells.

To our best knowledge, this is the first study to illustrate the biological interaction mechanisms between DDR pathways and endothelial cells toxic effect triggered by silica nanoparticles. Prior to undertaking in vitro toxicity experiments, the characterization of silica nanoparticles, which is essential for nanotoxicity studies, was performed by transmission electron microscope (TEM) and dynamic light scattering (DLS) measurements. To investigate the toxic effect mechanisms of endothelial cells induced by silica nanoparticles, we conducted a sequence of assessments including cellular uptake and morphology, cell viability, membrane integrity, intracellular ROS generation, oxidative damage, DNA damage, cell cycle arrest, apoptosis and necrosis after HUVECs exposure to silica nanoparticles for 24 h. We also measured the protein levels of Chk1, Cdc25c, cyclin B1/Cdc2 to analyze whether silica nanoparticles-induced endothelial cells toxic effect was through DDR via Chk1-dependent G2/M checkpoint signaling pathway.

## Materials and Methods

### Silica nanoparticles preparation and characterization

Silica nanoparticles were prepared using the Stöber method [25]. Briefly, 2.5 mL of tetraethylorthosilicate (TEOS) (Sigma, USA) was added to premixed ethanol solution (50 mL) containing ammonia (2 mL) and water (1 mL). The reaction mixture was kept at 40°C for 12 h with continuous stirring (150 r/min). The resulting particles were isolated by centrifugation (12,000 r/min, 15 min) and washed three times with deionized water and then dispersed in 50 mL of deionized water. The size and distribution of silica nanoparticles were performed by transmission electron microscope (TEM) (JEOL JEM2100, Japan) and ImageJ software. The hydrodynamic sizes and zeta potential of silica nanoparticles were examined by Zetasizer (Malvern Nano-ZS90, Britain). Suspensions of silica nanoparticles were dispersed by sonicator (160 W, 20 kHz, 5 min) (Bioruptor UDC-200, Belgium) before addition to culture medium in order to minimize their aggregation.

### Cell culture and exposure to silica nanoparticles

The primary human umbilical vein endothelial cells (HUVECs) line was purchased from the Cell Resource Center, Shanghai Institutes for Biological Sciences (SIBS, China). The cells were maintained in Dulbecco's Modified Eagle's Medium (DMEM) (Gibco, USA) supplemented with 10% fetal bovine serum (Gibco, USA), 100 U/mL penicillin and 100 µg/mL streptomycin, and cultured at 37°C in 5% CO_2_ humidified environment. For experiments, the cells were seeded in 6-well plates (except MTT assay using 96-well plates) at a density of 1×10^5^ cells/mL and allowed to attach for 24 h, then treated with silica nanoparticles suspended in DMEM of certain concentrations for another 24 h. Before use, the stock suspensions of silica nanoparticles were sonicated for 5 min. Controls were supplied with an equivalent volume of DMEM without silica nanoparticles. For all experiments, each group had five replicate wells. Data are expressed as means ± S.D. from three independent experiments (*p<0.05).

### Detection of silica nanoparticles uptake

LSCM detection: HUVECs were seeded at 1×10^4^ cells in 35 mm-diameter glass bottom cell culture dish and were cultured in DMEM as above. After 24 h of cell attachment, the cells were treated with Ruthenium (II) hydrate (Ru(phen)_3_^2+^) interior-labeled silica nanoparticles (50 µg/mL) for 24 h at 37°C in serum-free medium. These red fluorescent silica nanoparticles were prepared by a modified Stöber method and characterized as described before [26]. Cells were then washed several times with phosphate-buffered saline (PBS) and fixed with 4% paraformaldehyde at room temperature for 10 min. Cells were then washed 3 times with phosphate-buffered saline (PBS) and fixed with 4% paraformaldehyde at room temperature for 10 min. The cells were washed with 0.1% Triton X-100 three times and incubated with Phalloidin-FITC Actin-Tracker Green (Jiancheng, China) at room temperature for 30 min. The Actin-Tracker was dissolved in the mixture of 0.1% Triton X-100 and 3% bovine serum albumin (BSA) (Sigma, USA) for staining the actin filamentous skeleton. After that, the nucleus was stained with 5 µg/mL 4,6-diamidino-2-phenylindole (DAPI) (Sigma, USA) in PBS for 5 min. Cellular uptake were observed by a laser scanning confocal microscopy (LSCM) (Leica TCS SP5, Germany).

TEM detection: After HUVECs incubated for 24 h with silica nanoparticles (50 µg/mL), the cells were washed with PBS and then centrifuged at 2000 r/min for 10 min. The supernatants were removed. The cell pellets were fixed in a 0.1 M PBS solution containing 2.5% glutaraldehyde and 4% paraformaldehyde for 3 h. They were then washed with 0.1 M PBS, embedded in 2% agarosegel, postfixed in 4% osmium tetroxide solution for 1 h, washed with distilled water, stained with 0.5% uranyl acetate for 1 h, dehydrated in a graded series of ethanol (30%, 60%, 70%, 90%, and 100%), and embedded in epoxy resin. The resin was polymerized at 60°C for 48 h. Ultrathin sections obtained with a ultramicrotome were stained with 5% aqueous uranyl acetate and 2% aqueous lead citrate, air dried, and imaged under a transmission electron microscope (TEM) (JEOL JEM2100, Japan).

### Assessment of cytotoxicity

Cultured HUVECs were treated with various concentrations (25, 50, 75, and 100 µg/mL) of silica nanoparticles for 24 h. Cell morphology was observed by optical microscope (Olympus IX81, Japan).

The cell viability was measured using the 3-(4, 5-dimethylthiazol-2-yl)-2, 5-diphenyltetrazolium bromide (MTT) reduction assay. MTT assay is the most common employed for the detection of cytotoxicity or cell viability following exposure to toxic substances. MTT is a water soluble tetrazolium salt, which is converted to an insoluble purple formazan by cleavage of the tetrazolium ring by succinate dehydrogenase within the mitochondria. The formazan product is impermeable to the cell membranes and therefore it accumulates in healthy cells. The absorbance of formazan was measured at 492 nm using a microplate reader (Themo Multiscan MK3, USA).

The lactate dehydrogenase leakage assay (LDH), which is based on the measurement of lactate dehydrogenase activity in the extracellular medium, was determined using a commercial LDH Kit (Jiancheng, China) according to the manufacturer's protocols. The loss of intracellular LDH and its release into the culture medium is an indicator of irreversible cell death due to cell membrane damage. After HUVECs treated with different concentrations (25, 50, 75, and 100 µg/mL) of silica nanoparticles for 24 h, the supernatants were collected for LDH measurement. 100 µL cell medium was used for LDH activity analysis and the absorbance at 440 nm was measured by a UV-visible spectrophotometer (Beckman DU-640B, USA).

### Apoptosis and necrosis

Apoptosis in endothelial cells was measured using the Annexin V-propidium iodide (PI) apoptosis detection kit (KeyGen, China). The kit contains Annexin V conjugated to the flurochrome FITC. This complex displays a high affinity to the membrane phospholipid phosphatidylserine, which undergoes externalization in the earlier stages of apoptosis. To distinguish early apoptotic cells from dead cells resulted from late apoptosis or necrosis, the vital dye PI was used. In this way, FITC negative and PI negative were designated as live cells in the lower left quadrant; FITC positive and PI negative as apoptotic cells in the upper left quadrant; FITC positive and PI positive as necrotic cells in the upper right quadrant; and FITC negative and PI positive as large nuclear fragments in the lower right quadrant [27]. HUVECs were exposed to silica nanoparticles for 24 h, washed with PBS three times and trypsinized. After centrifugation at 1000 rpm, the cell pellet was washed with PBS once and incubated with 5 µL Annexin V-FITC for 15 min, which was followed by staining with 5 µL PI. Then, the samples were diluted with 500 µL binding buffer and analyzed with a flow cytometer (Becton Dickinson, USA), and at least 1×10^4^ cells were counted for each sample.

### Intracellular ROS measurement

The cytotoxicity effects might occur through the induction of oxidative stress and apoptosis with possible involvement of overproduction of reactive oxygen species (ROS). In this regard, the production of intracellular ROS was measured by flow cytometry using the 2′, 7′-dichlorofluorescein diacetate (DCFH-DA) (Beyotime, China) as an oxidation-sensitive probe. Briefly, 10 mM DCFH-DA stock solution was diluted 1000-fold in cell culture medium without serum or other additive to yield a 10 µM working solution. After the exposure of HUVECs to series dosages (25, 50, 75, and 100 µg/mL) of silica nanoparticles for 24 h, the cells in 6-well plates were washed twice with PBS and incubated in 2 mL working solution of DCFH-DA at 37°C in dark for 30 min. Then the cells were washed twice with cold PBS and resuspended in the PBS for analysis. Fluorescent intensity and percentage of positive cells were measured by flow cytometry (Becton-Dickison, USA). For each sample, at least at least 1×10^4^ cells were collected.

### Assessment of oxidative damage

In addition to the analysis of cytotoxicity and ROS levels, the malondialdehyde (MDA) content was measured as an end product of lipid peroxidation. The defense systems against free radical attack were assessed by the measurement of both the activities of superoxide dismutase (SOD) and glutathione peroxidase (GSH-Px). After HUVECs exposure to different concentrations (25, 50, 75, and 100 µg/mL) of silica nanoparticles for 24 h, washed once with ice-cold PBS, and lysed in ice-cold RIPA lysis buffer containing 1 mM phenylmethylsulphonyl fluoride (PMSF) (DingGuo, China) and phosphatase inhibitor for 30 min. After centrifuging the lysates at 12,000 rpm, 4°C for 10 min, the supernatants were collected for measurements of the production of MDA, the activities of SOD and GSH-Px. All examinations were carried out using commercially available kits (Jiancheng, China) according to the manufacturer's instructions. The protein concentrations of these extracts were determined by performing the bicinchoninic acid (BCA) protein assay (Pierce, USA).

### Detection of mitochondrial membrane potential (MMP)

MMP was detected by using 5,5′,6,6′-tetrachloro-1,1′,3,3′-tetraethylbenzimi dazo-lylcarbocyanide iodine (JC-1) (Sigma, USA). This probe can selectively enter into mitochondria and reversibly change color from red to green as the membrane potential decreased. The ratio of green to red expresses the change of MMP. Cells were treated with silica nanoparticles (25, 50, 75, and 100 µg/mL) for 24 h. After washing with PBS, the cells were incubated with 10 µg/mL working solution of JC-1 for 20 min. Then the cells were washed with PBS twice and analyzed by flow cytometry (Becton-Dickison, USA). The green fluorescence intensity was determined at an excitation wavelength of 488 nm and an emission wavelength of 525 nm, whereas the red fluorescence intensity determined at an excitation wavelength of 488 nm and an emission wavelength of 590 nm. For each sample, at least at least 1×10^4^ cells were collected.

### Comet assay

Comet assay, also known as single cell gel electrophoresis (SCGE), is a visual and sensitive technique for measuring DNA breakage in individual mammalian cells. The DNA damage induced by silica nanparticles was performed by Single cell gel electrophoresis kit (Biolab, China). HUVECs were collected and resuspended in PBS. 20 µL of the cells suspension and 80 µL of low melting agarose were mixed and 80 µL of the suspension pipetted onto a comet-slide. The slides were placed flat in dark at 4° C for 10 min for the mixture to solidify. Then the slides were placed in pre-chilled lysing solution at 4° C for 2 h. Slides were removed from lysing solution, tapped on a paper towel to remove any excess lysis solution and immersed in alkaline solution for 45 min in dark at room temperature. The slides were washed twice for 5 min. Then the slides were electrophoresed at low voltage (300 mA, 25 V) for 30 min. Slides were removed from the electrophoresis unit after the designated time, tapped to remove excess buffer at room temperature. Subsequently, the air-dried slides were stained with DNA-binding dye propidium iodide (PI) and evaluated under a fluorescence microscope (Olympus IX81, Japan). To prevent additional DNA damage, all the steps described above were conducted under dimmed light or in the dark. The data were analyzed by CASP software based on 100 randomly selected cells per sample. The percentage of tail DNA, tail length and Olive tail moment (OTM) were selected as indicators of DNA damage.

### Cell cycle arrest

The cell cycle detection kit (KeyGen, China) was used to determine the DNA index (DI) and cell-cycle phase distributions. The method involved dissolving of the cell membrane lipids, eliminating the cell cytoskeleton with trypsin, digesting the cellular RNA and stabilizing the chromatin with spermine. HUVECs were exposed to various concentrations of silica nanoparticles for 24 h, washed with PBS three times and trypsinized. After centrifuged at 1000 rpm for 5 min, the cells were washed one time in PBS and fixed in 70% ethanol for at least 24 h. The fixed cells were washed twice with PBS and treated with 100 µL RNase A at 37°C for 30 min. Finally, the cells were stained with 400 µL propidium iodide and incubated in dark for 30 min. A total of at least 1×10^4^ cells for each sample were analyzed by flow cytometry (Becton Dickinson, USA).

### Western blot analysis

To analyze whether silica nanoparticles influence the expression of G2/M DNA damage checkpoint regulators, we measured the protein levels of Chk1, Cdc25c, cyclin B1/Cdc2 in HUVECs by western blot analysis. Total cellular protein extracts were determined by performing the bicinchoninic acid (BCA) protein assay (Pierce, USA). The equal amounts of lysate proteins (40 µg) were loaded onto SDS-polyacrylamide gels (12% separation gels) and electrophoretically transferred to polyvinylidene fluoride (PVDF) membranes (Millipore, USA). After blocking with 5% nonfat milk in Tris-buffered saline (TBS) containing 0.05% Tween-20 (TBST) for 1 h at room temperature, the membrane was incubated with Chk1, Cdc25C, Cdc2, cyclin B1 [1∶1000, rabbit antibodies, Cell Signaling Technology (CST), USA] overnight at 4°C, washed with TBST, and incubated with a horseradish peroxidase-conjugated anti-rabbit Ig G secondary antibody (CST, USA) for 1 h at room temperature. After washed three times with TBST, The antibody-bound proteins were detected using the ECL chemiluminescence reagent (Pierce, USA).

Densitometric analysis of the western blots was performed using Image Lab™ Software (Bio-Rad Version 3.0, USA). The relative values of the samples were measured by normalising all data to the respective control samples of each experiment.

### Statistical analysis

Data were expressed as mean ± S.D. and significance was determined by using one-way analysis of variance (ANOVA) followed by least significant difference (LSD) test to compare the differences between groups. Differences were considered significant at *p*<0.05.

## Results

### Characterization of silica nanoparticles

**Figure 1:**
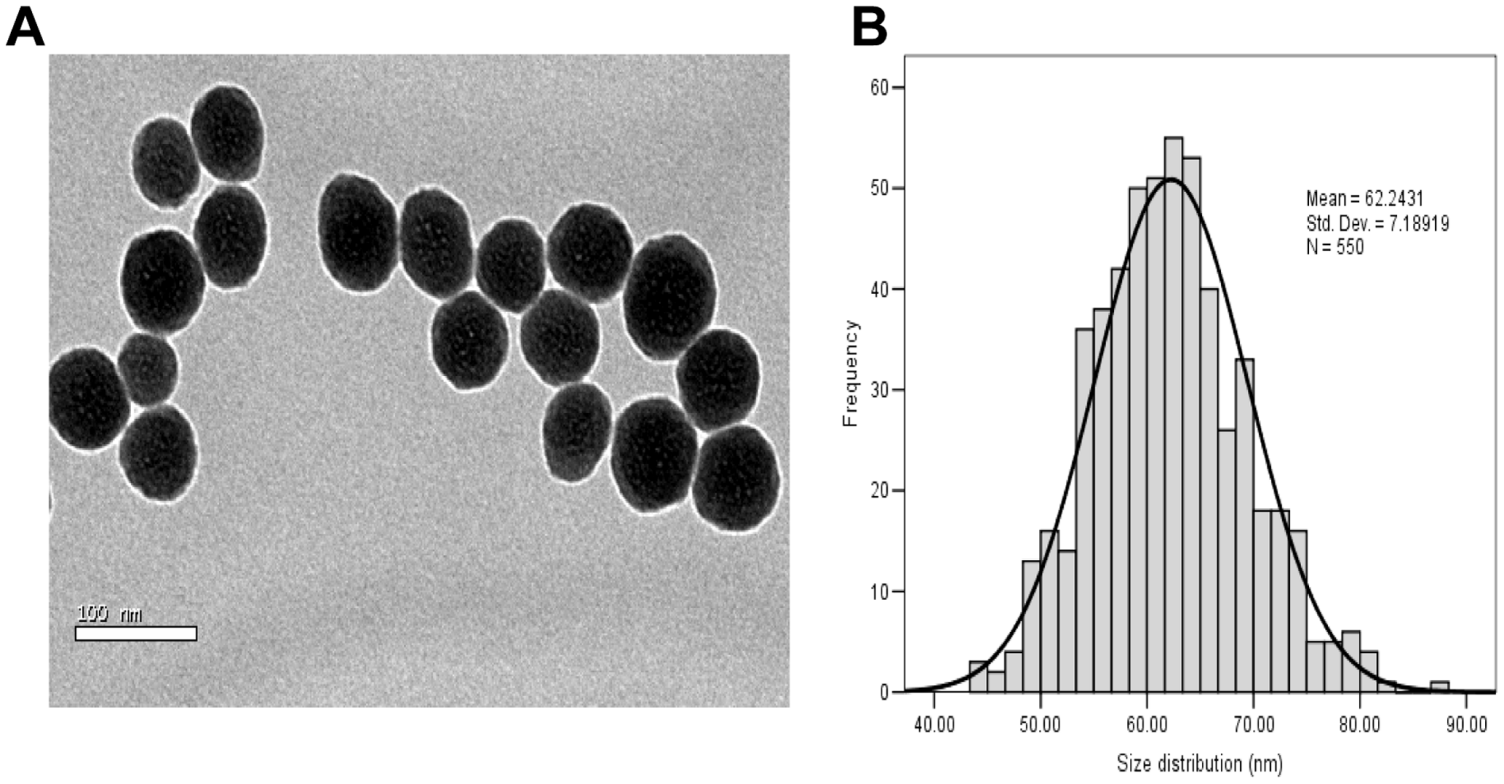
Characterization of silica nanoparticles. (A) Transmission electron microscopy image: TEM images of silica nanoparticles had a spherical shape with the average diameter of 62 nm. (B) Size distribution: The size distribution measured by ImageJ software showed approximately normal distribution.

As shown in Figure 1A, the TEM images of silica nanoparticles had a spherical shape with the average diameter of 62 nm. The size distribution measured by ImageJ software showed approximately normal distribution (Figure 1B). The hydrodynamic sizes of silica nanoparticles were measured in distilled water as stock media and in DMEM as culture media at different time point to reflect their dispersion by polydispersity index (PDI) (Table 1). The silica nanoparticles exhibited very good monodispersity in DMEM. Zeta potentials provide quantitative information on the stability of the particles. Silica nanoparticles tested in our study had the absolute value of zeta potentials is higher than 30 mV. It is well documented that the particles are more likely to remain dispersed if the absolute value of zeta potential is higher than 30 mV [28]. Our results showed that the silica nanoparticles in culture medium possessed uniform shape along with relatively favourable dispersibility.

**Table 1:** Hydrodynamic size and Zeta potential of silica nanoparticles in dispersion media.

	Distilled water			DMEM		
Time (h)	Diameter (nm)	PDI	Zeta potential (mV)	Diameter (nm)	PDI	Zeta potential (mV)
1	108.03±0.61	0.11±0.01	−40.33±6.47	106.03±0.93	0.11±0.02	−35.27±2.10
3	106.80±0.63	0.10±0.01	−39.13±5.26	105.83±0.90	0.10±0.02	−36.77±2.40
6	105.60±1.22	0.07±0.02	−41.43±3.29	107.27±0.93	0.13±0.01	−36.53±0.64
12	104.97±0.60	0.10±0.02	−44.10±1.30	104.23±1.17	0.08±0.01	−34.37±2.75
24	104.87±0.64	0.08±0.02	−46.33±3.13	104.43±0.21	0.10±0.01	−38.10±0.46

Data are expressed as means ± S.D. from three independent experiments.

### Cellular uptake of silica nanoparticles

**Figure 2:**
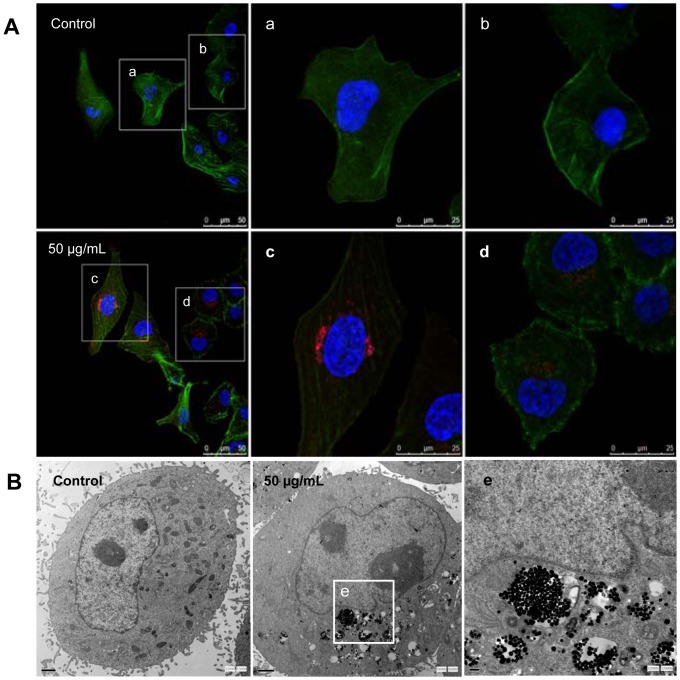
Subcellular localization of silica nanoparticles. (A) LSCM images of HUVECs after incubation for 24 h with Ruthenium (II) hydrate labeled silica nanoparticles (50 µg/mL, red) of size 62 nm. The cell skeleton was stained with Phalloidin-FITC (green), and the cell nucleus with 4,6-diamidino-2-phenylindole (DAPI; blue). (B) TEM images of HUVECs exposed to silica nanoparticles for 24 h. Both TEM and LSCM results showed that the silica nanoparticles were internalized into cells compared to control group.

Since the subcellular localization may play an important role in silica nanoparticles-induced biological effects, we examined the HUVECs uptake of Ru(phen)_3_^2+^-labeled silica nanoparticles by LSCM. Merged confocal microscopic images of HUVECs (Figure 2A) showed that the fluochrome-labeled silica nanoparticles were internalized into cells compared to control group after 24 h exposure, which was consistent with TEM images (Figure 2B).

### Cytotoxicity of HUVECs induced by silica nanoparticles

**Figure 3:**
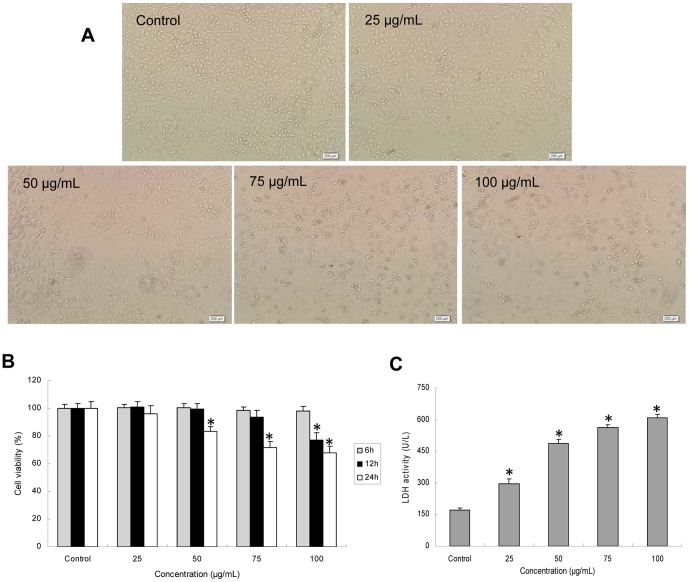
Cytotoxicity of HUVECs induced by silica nanoparticles. (A) Morphological changes of HUVECs after exposure to silica nanoparticles for 24 h. Cell density reduction, irregular shape and cellular shrinkage were observed by optical microscope. (B) Cell viability of HUVECs treated with silica nanoparticles was measured by MTT assay after 6 h, 12 h, 24 h exposure. (C) LDH leakage of HUVECs exposed to viarous concentrations of silica nanoparticles for 24 h. The results indicated that silica nanoparticles induced cytotoxicity in a dose- and time-dependent manner. Data are expressed as means ± S.D. from three independent experiments (*p<0.05).

To evaluate the possible toxicity of silica nanoparticles on endothelial cells, cellular morphology and cell viability were determined after exposing HUVECs to silica nanoparticles for 24 h. With the dosages (25, 50, 75, and 100 µg/mL) increasing, the morphological changes of HUVECs became more and more obviously. Cell density reduction, irregular shape and cellular shrinkage were observed (Figure 3A). Compared with control group, the cell density in 100 µg/mL treated group was obviously reduced after 24 h exposure. Change of cellular morphology was directly reflected with cell viability. As indicated in Figure 3B, viability of HUVECs induced by silica nanoparticles showed no significant change as early as at 6 h. As time passed, cell viability was decreased remarkably in 100 µg/mL treated group at 12 h. Up to 24 h, the cell viability in 50 µg/mL treated group decreased to 83.49%, which was significantly lower than that of control. In addition, the MTT results were strongly in accordance with the increased membrane damage measured by LDH release (Figure 3C). Significant difference of LDH release was observed between treated groups and control group after HUVECs exposure to silica nanoparticles for 24 h. Our results indicated that silica nanoparticles induced cytotoxicity in a dose- and time-dependent manner.

### Apoptosis and necrosis of HUVECs induced by silica nanoparticles

**Figure 4:**
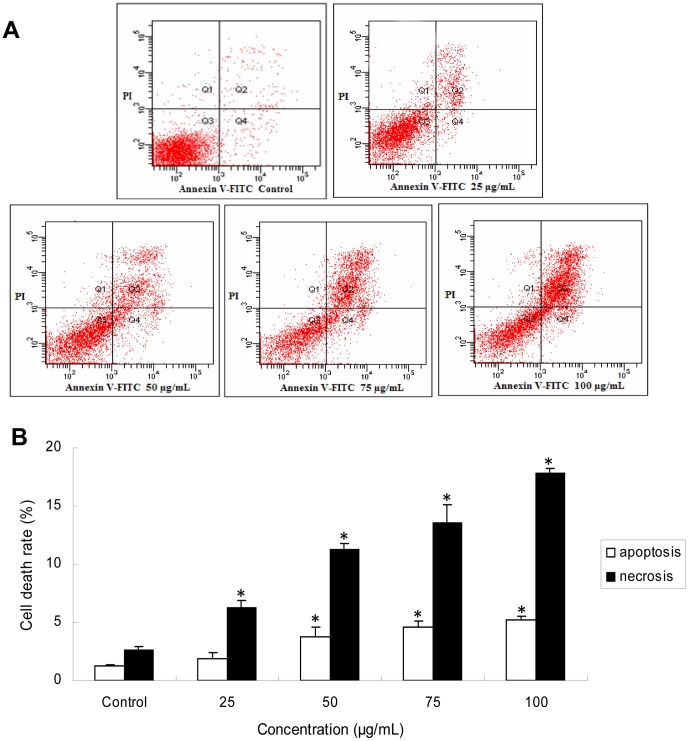
Apoptosis of HUVECs after exposure to silica nanoparticles for 24 h. (A) Apoptotic and necrotic populations of cells double-stained with PI- and FITC-labled Annexin V were depicted by flow cytometry. FITC negative and PI negative were designated as live cells in the lower left quadrant; FITC positive and PI negative as apoptotic cells in the upper left quadrant; FITC positive and PI positive as necrotic cells in the upper right quadrant; and FITC negative and PI positive as large nuclear fragments in the lower right quadrant. (B) HUVECs exposure to silica nanoparticles caused increase of both necrosis and apoptosis rate. The apoptosis rate was much lower than the necrosis rate. Data are expressed as means ± S.D. from three independent experiments (*p<0.05).

To further analyze the cell death caused by silica nanoparticles, apoptosis and necrosis in HUVECs were measured by flow cytometry. As shown in Figure 4, after 24 h of exposure, the apoptotic rate was significant increased in 50 µg/mL treated group compared to that of control group. While the necrosis rate was significant elevated in as low as 25 µg/mL. The apoptosis rate was much lower than necrosis rate induced by silica nanparticles. Our data indicated that the silica nanoparticles could cause both apoptosis and necrosis. The membrane damage and necrosis were mainly responsible for cell death caused by silica nanoparticles.

### Intracellular ROS generation induced by silica nanoparticles

**Figure 5:**
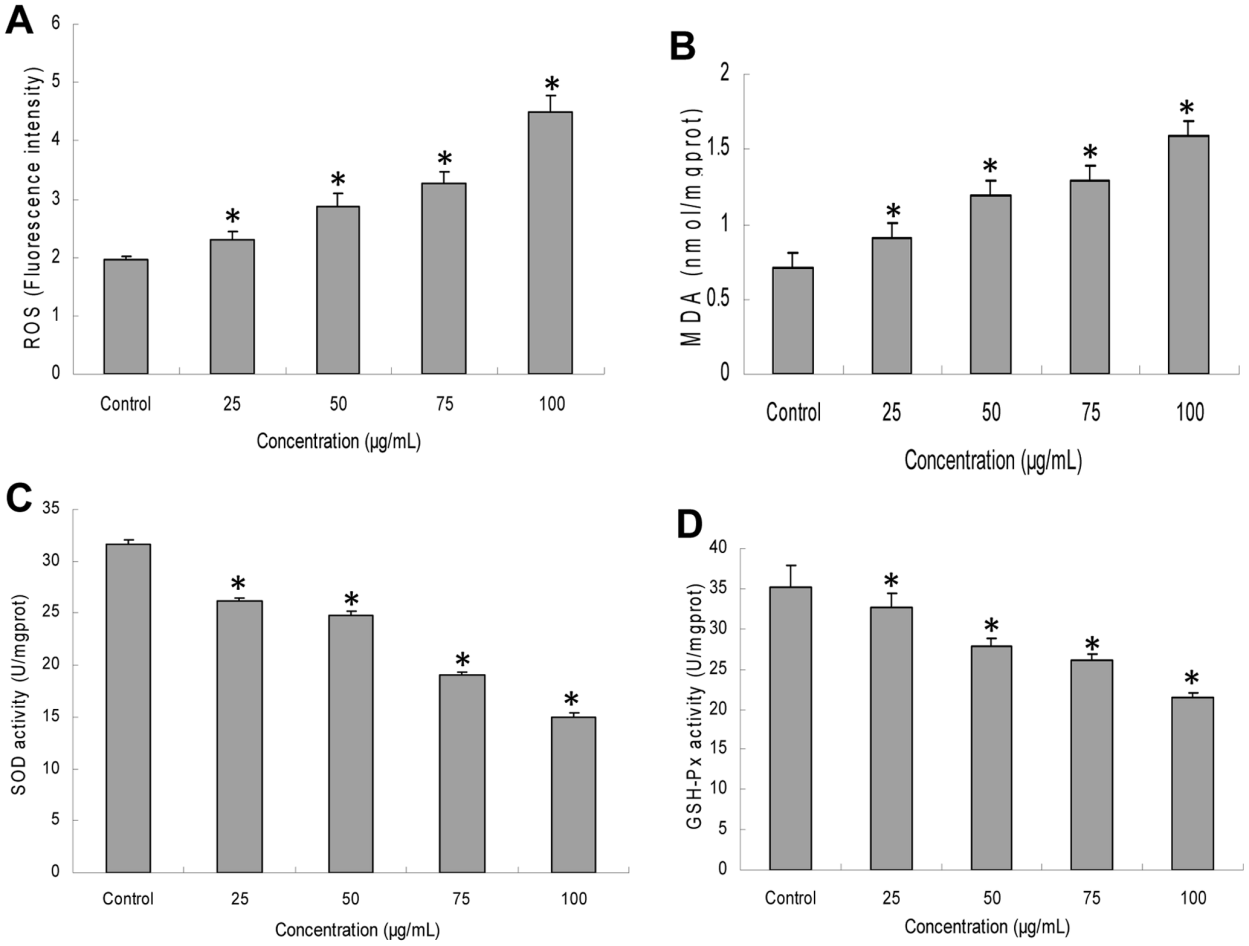
Oxidative stress and oxidative damage induced by silica nanoparticles on HUVECs. The intracellular levels of ROS and MDA were obviously increased (A, B). While SOD and GSH-Px levels were decreased significantly with a dose-dependent way (C, D). Silica nanoparticles-induced ROS generation caused oxidative damage followed by the production of MDA as well as the inhibition of SOD and GSH-Px. Data are expressed as means ± S.D. from three independent experiments (*p<0.05).

To get a closer insight into cytotoxicity induced by silica nanoparticles, we measured the generation of ROS through fluorescence intensity of dichlorofluorescein (DCF). As shown in Figure 5A, after HUVECs exposure to silica nanoparticles for 24 h, the intracellular ROS levels of all treated groups were increased gradually. In 100 µg/mL treated group, the fluorescence intensity was significantly elevated (2.3-fold much higher than that of control). Our results demonstrated that silica nanoparticles induced intracellular ROS generation increased in a dose-dependent manner.

### Oxidative damage triggered by silica nanoparticles

The generation of intracellular ROS could cause oxidative damage. Therefore, we measured the production of MDA as well as the activities of SOD and GSH-Px. Figure 5B shown that the intracellular level of MDA in HUVECs exposed to silica nanoparticles for 24 h was significantly increased compared to that of control group. In contrast, the levels of the SOD and GSH-Px were decreased significantly with a dose-dependent way (Figure 5C and Figure 5D). The results revealed that silica nanoparticles-induced ROS generation in HUVECs caused oxidative damage followed by the production of MDA and the inhibition of SOD and GSH-Px.

### Changes of mitochondrial membrane potential induced by silica nanoparticles

**Figure 6:**
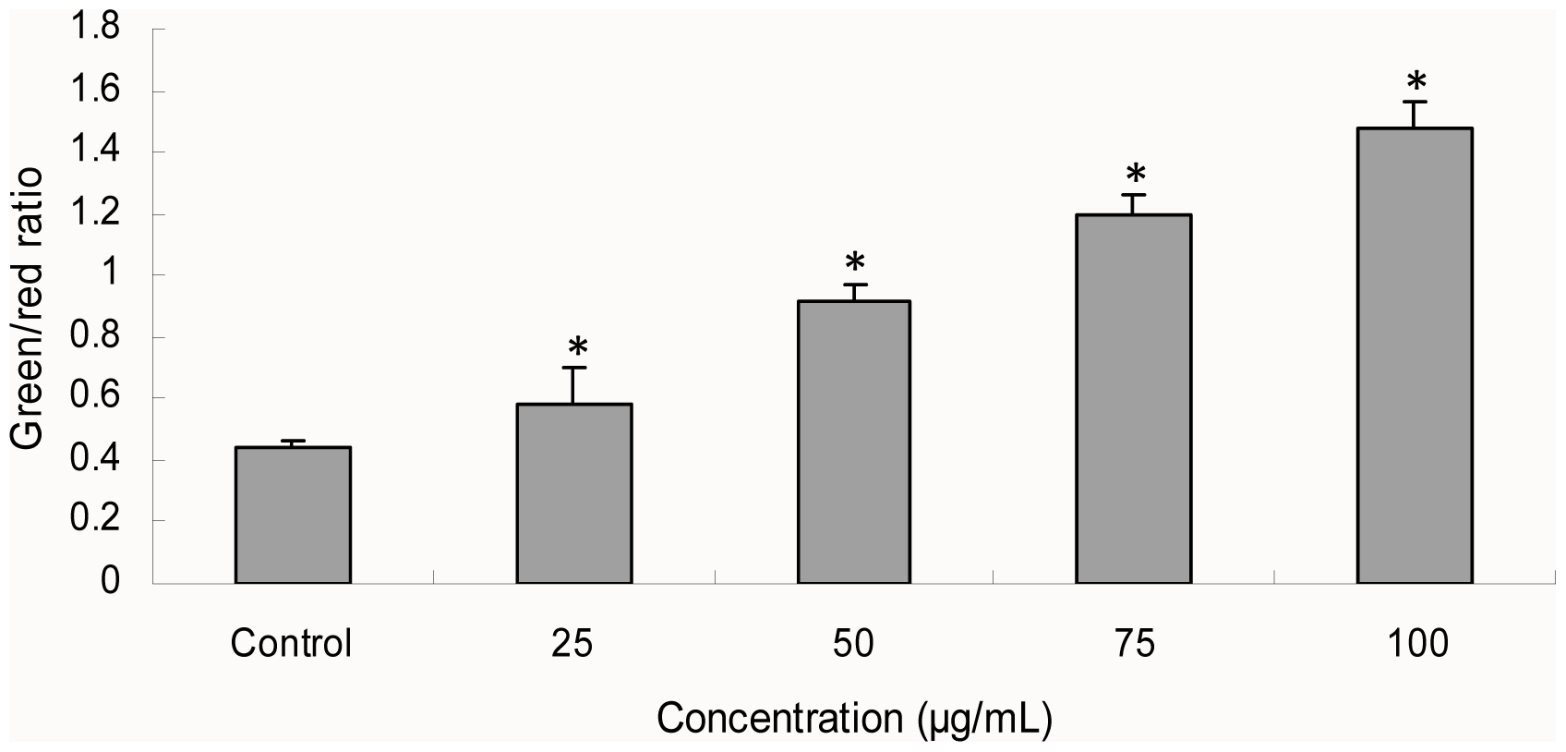
Mitochondrial membrane potential changes after silica nanoparticles exposure for 24 h detected with JC-1 probe by flow cytometry. The green/red fluorescence intensity ratio was used to express the changes of MMP and the increased ratio indicates decrease of MMP. Data are expressed as means ± S.D. from three independent experiments (*p<0.05).

The MMP was determined with JC-1 probe by flow cytometry (Figure 6). The green/red fluorescence intensity ratio was used to express the changes of MMP and the increased ratio indicates decrease of MMP. Our results showed the ratio elevated with the increasing of silica nanoparticles exposure concentrations indicating the MMP changes were concentration dependent. The MMP of all the silica nanoparticles treated groups at 24 h have significant differences compared with control group.

### DNA damage of HUVECs induced by silica nanoparticles

**Figure 7:**
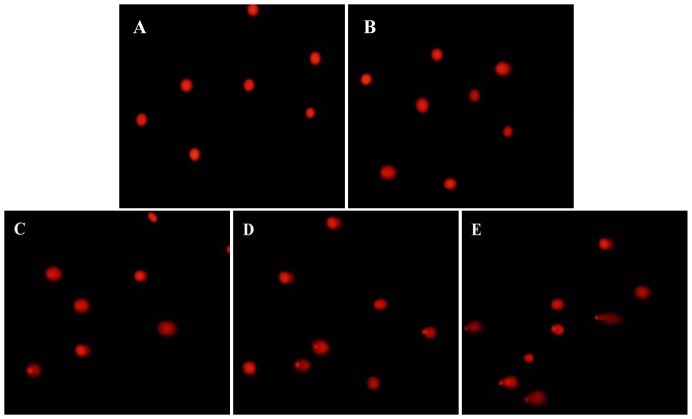
DNA damage of HUVECs after exposed to silica nanoparticles for 24 h determined by comet assay. (A) Control group, (B) 25 µg/mL treated group, (C) 50 µg/mL treated group, (D) 75 µg/mL treated group, (E) 100 µg/mL treated group. More severe DNA injury was reflected by larger area of the comet tail. The DNA damage caused by silica nanoparticles was getting more serious with the dosages increasing. The magnification was 200× by fluorescence microscope.

To investigate the mechanisms of apoptosis induced by silica nanoparticles, DNA damage was measured by comet assay. As shown in Figure 7 and Table 2, HUVECs exposure in 25 µg/mL treated group showed no significant difference in DNA damage. With the dosages increasing, the degree of DNA damage involving the percentage of tail DNA, tail length and Olive tail moment (OTM) were significantly elevated. Our data indicated that the DNA damage caused by silica nanoparticles was getting more serious with the dosages increasing.

**Table 2:** DNA damage of HUVECs induced by silica nanoparticles.

	DNA damage		
Groups	Tail DNA (%)	Tail Length (µm)	Olive Tail Moment
Control	0.40±0.18	2.75±0.46	0.05±0.02
25 µg/mL	1.72±0.62	3.56±0.73	0.40±0.10
50 µg/mL	15.99±2.56*	14.25±4.98*	6.41±2.62*
75 µg/mL	24.76±6.88*	23.88±9.33*	11.60±3.99*
100 µg/mL	34.21±5.23*	31.80±5.53*	15.99±3.65*

Data are expressed as means ± S.D. from three independent experiments (*p<0.05).

### Cell cycle arrest of HUVECs induced by silica nanoparticles

**Figure 8:**
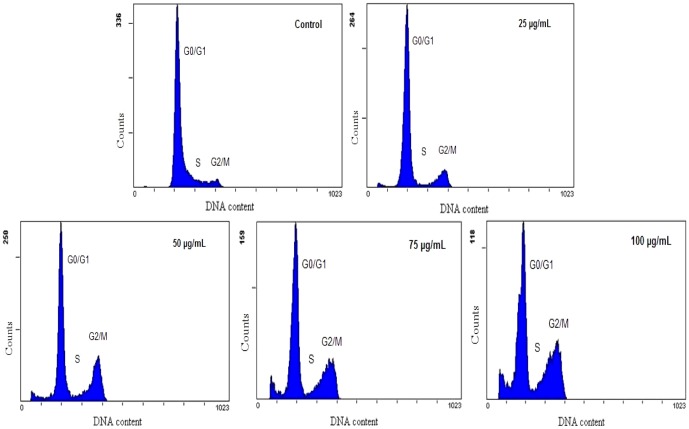
Cell cycle arrest of HUVECs induced by silica nanoparticles. After exposure to various concentrations of silica nanoparticles for 24 h, flow cytometry were used to determine the cell cycle distribution of HUVECs. The images showed that cell cycle was arrested in G2/M phase. The percentage of cells in G2/M phase increased progressively in a dose-dependent manner, while in G0/G1 and S phase the percentage of cells declined irregular.

The relevant checkpoints could arrest cell cycle at certain stage as response to DNA damage. Thus, we measured the cell cycle arrest by flow cytometry. As shown in Figure 8 and Table 3, the cell cycle was arrested in G2/M phase. The percentage of cells in G2/M phase increased progressively in a dose-dependent manner, while in G0/G1 and S phase the percentage of cells declined irregular. At the dosages up to the highest concentration (100 µg/mL), the quantitative analysis showed that HUVECs exposure to silica nanoparticles resulted in a significant 5.1-fold increasing of G2/M phase compared to that of control group. Therefore, our data revealed that the silica nanoparticles induced G2/M arrest in HUVECs triggered by oxidative DNA damage.

**Table 3:** Cell cycle arrest of HUVECs induced by silica nanoparticles.

	Distribution of cell cycle		
Groups	G0/G1	S	G2/M
Control	67.19±0.77	25.97±0.90	6.84±0.99
25 µg/mL	77.70±1.88*	11.68±1.82*	10.62±0.85
50 µg/mL	59.54±2.14*	19.07±1.84*	21.39±1.41*
75 µg/mL	57.41±1.37*	15.10±1.83*	27.49±0.66*
100 µg/mL	59.91±3.21*	5.48±1.14*	34.62±3.14*

Data are expressed as means ± S.D. from three independent experiments (*p<0.05).

### Chk1-dependent G2/M checkpoint pathways activated by silica nanoparticles

**Figure 9:**
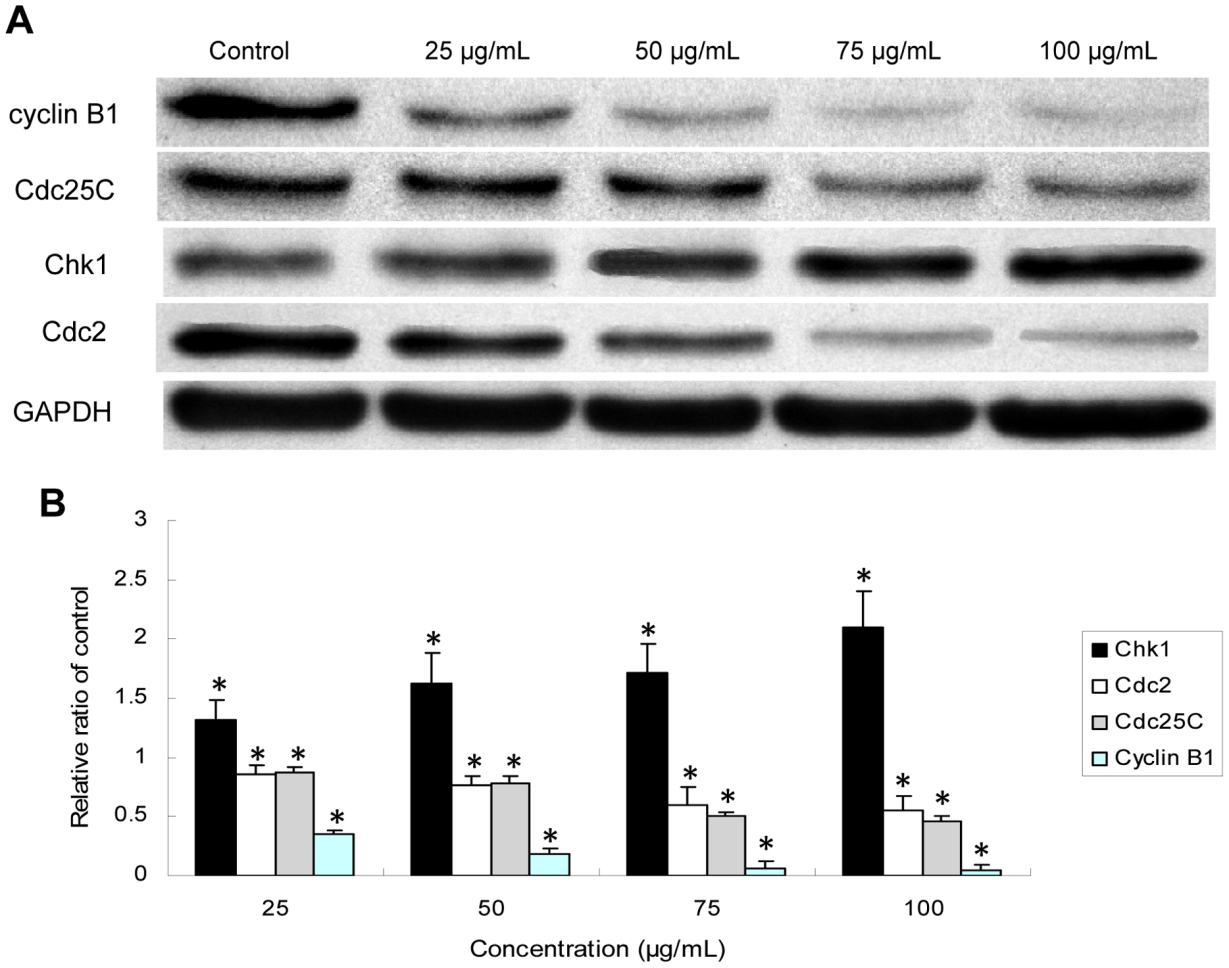
Effects of silica nanoparticles on G2/M DNA damage checkpoint signaling pathway . (A) Effect of silica nanoparticles on the expression of Chk1, Cdc25C, cyclin B1, Cdc2. GAPDH was used as an internal control to monitor for equal loading. (B) Relative densitometric analysis of the proteins bands was performed and presented. Silica nanoparticles induced G2/M arrest through the upregulation of Chk1 and the downregulation of Cdc25C, cyclin B1/Cdc2. Data are expressed as means ± S.D. from three independent experiments (*p<0.05).

To better understand the G2/M arrest signaling pathway, we examined the expression of G2/M checkpoint regulators in HUVECs by western blot analysis. As shown in Figure 9, the expression of Chk1 was obviously increased after HUVECs exposed to silica nanoparticles for 24 h, whereas the expression of Cdc25C, Cdc2 and cyclin B1 were marked decreased compared to that of control group. Our data demonstrated that the G2/M regulation pathway was affected or disturbed by silica nanoparticles through the activation of Chk1 and the inhibition of Cdc25C and cyclin B1/Cdc2. The downregulation of cyclin B1/Cdc2 resulted in a directly arrest of G2/M phase in HUVECs.

## Discussion

Nanoscience has matured significantly during the last decade as it has transitioned from bench top science to applied technology [29]. Presently, silica nanoparticles are widely used in biomedical applications as promising carriers for drug delivery or gene therapy. Thus, the endothelial cells could be primarily exposed to silica nanoparticles by intravenous administration. However, biological or cellular responses to silica nanoparticles are still poorly understood. DNA damage response (DDR) involved the sensing of DNA damage followed by transduction of the damage signal plays an important role in the network of cellular pathways. To our best knowledge, the possibility mechanisms of DDR pathways triggered by silica nanoparticles caused the toxic effect of endothelial cells has not been investigated. Our findings demonstrated that direct exposure of HUVECs to silica nanoparticles induced DDR leading to activate the Chk1-dependent G2/M checkpoint signaling pathway, resulted in a series of endothelial cells toxic effect.

Currently, cell uptake of nanoparticles is an important issue in designing suitable cell-tracking and drug-carrier nanomaterials systems [30]. LSCM and TEM results showed that silica nanoparticles were internalized into endothelial cells after 24 h exposure (Figure 2). In addition, our previous study confirmed that the silica nanoparticles could internalized into the cells and dispersed in cytoplasm and deposited inside mitochondria [25]. To gain closer mechanistic insight into silica nanparticles-induced biological effects, we measured the cellular morphology, cell viability and membrane integrity as cytotoxicity indicators in HUVECs. Exposure to cytotoxic agents can affect cellular morphology, which is directly reflecting cell injuries. Firstly, we examined the morphology of HUVECs exposure to silica nanoparticles for 24 h by optical microscopy (Figure 3A). Cell density reduction, irregular shape as well as cellular shrinkage were observed. Changes in cellular morphology have been considered as a direct indicator in assessing cytotoxicity [27]. To confirm and analyze this observation, cell viability and LDH release were measured (Figure 3B and 3C). Our data revealed that the silica nanoparticles-induced cytotoxicity increased in a dose- and time-dependent manner.

It has been confirmed that the LDH release is an indicator of necrosis due to cell membrane damage [31]. To further analyze the cell death caused by silica nanoparticles, apoptosis and necrosis in HUVECs were measured (Figure 4A). In accordance with LDH results, a significant increase of necrosis rate was noted at the concentrations (25, 50, 75, and 100 µg/mL) of silica nanoparticles, while the apoptosis rate was much lower than necrosis (Figure 4B). Rapid-acting metabolic poisons and strong physical stress can cause necrosis accompanied with membrane damage. In contrast, apoptosis is a slow-acting form of cell death accompanied with an energy-dependent sequence of events, resulted in fragmenting nuclei and cytoplasmic organelles ultimately, thus, the membrane damage is not a primary event of apoptosis [32]. In this study, we found that HUVECs exposure to silica nanoparticles also caused apoptosis (Figure 4). Similar results were obtained from Liu and coworker who suggested that the endothelial cells exposure to silica nanoparticles could cause apoptosis [18]. Endothelial cells apoptosis was considered as a major determinant of atherothrombosis [33]. To investigate the possible mechanisms of apoptosis induced by silica nanoparticles, intracellular ROS, MDA and antioxidant activities including SOD and GSH-Px were measured (Figure 5). The generation of intracellular ROS caused oxidative damage followed the production of lipid peroxidation and the inhibition of antioxidant activities. Generally, the oxidative stress produced by nanoparticles was considered to be one of the important aspects associated with nanotoxicity [34]. It was reported that silica nanoparticles showed stable surface radicals and sustained release of Hydroxyl radical (·OH). The ·OH radical is the most reactive ROS and triggers extensive cellular damage. ROS generated by the silica nanoparticles surface can induce cell membrane damage via lipid peroxidation that may subsequently lead to increased cellular permeability [35], [36]. Oxidative stress is the result of an imbalance in the pro-oxidant/antioxidant homeostasis. It is well-known that extensive increase in the ROS production exceeds the capacity of antioxidant mechanisms causing injury to lipids, proteins and DNA [37]. Induction of oxidative stress by silica nanoparticles have been observed in various cell types [38], [39], [40]. It may also due to their direct or indirect effects to some organelles of nanoparticles which entered the cells. These organelles, such as mitochondria, are the main sources of cellular ROS and the basis of the ROS metabolism [41], [42]. Oxidative stress induced membrane lipid peroxidation could occur both in vitro and in vivo, especially in membranes of highly metabolically active mitochondria [42]. In the present study, the mitochondrial membrane potential decreased obviously in a dose-dependent manner (Figure 6). Excess ROS production produced by silica nanoparticles exposure is one of the factors leading to the collapse of mitochondrial membrane potential [25]. Since the maintenance of ROS homeostasis depended on the respiratory chain and the membrane potential, oxidative damage may occur due to the decreasing of membrane potential [41], [43].

In addition, DNA damage could be mediated by oxidative stress depending on the balance between ROS production and antioxidant status [44]. The high surface area associated with nanoparticles can promote the generation of ROS, resulting in oxidative DNA damage [45]. Our previous study confirmed that silica nanoparticles induced ROS directly lead to DNA damage and cell cycle arrest [19]. The cellular response to DNA damage, commonly known as DDR, encompasses multiple repair mechanisms and checkpoint responses that can delay cell cycle progressing or modulate DNA replication [21]. It had been reported that silica nanoparticles could induce DDR, mutagenic effects and cell cycle arrest in various non-endothelial cell lines [46], [47], [48]. However, whether the toxic effect of endothelial cells is associated with DDR pathways has not been reported. In the present study, our results showed that the degree of DNA damage including the percentage of tail DNA, tail length and Olive tail moment (OTM) were significantly aggravated in a dose-dependent manner (Figure 7 and Table 2). Moreover, our data indicated that the silica nanoparticles inhibited HUVECs proliferation by inducing G2/M arrest (Figure 8 and Table 3). In response to DNA damage, cells launch elegant networks of genome surveillance mechanisms, called cell cycle checkpoints, to detect and repair damaged DNA to maintain the genome stability [49]. When cells have DNA damage to be repaired or DNA replication is not complete, these checkpoints will arrest cell cycle at one of the G0/G1, S or G2/M phase. The G2/M phase has played an important role in mitotic processes. G2/M DNA damage checkpoint serves to prevent the cell from entering mitosis (M-phase) with genomic DNA damage [50]. This kind of cell cycle delay could offer more time for the repair of DNA damage and avoid gene mutation [51]. However, when the DNA injuries of cells were so severe that exceed the cellular repair capacity, apoptosis would occur. Cell cycle checkpoints are pivotal mechanisms safeguarding genome stability. Cells that harbor defects in checkpoints are predisposed to genome instability and neoplastic transformation [52]. Therefore, it is necessary to further investigate the cell signaling pathway of silica nanoparticles-induced G2/M arrest.

In the current study, we confirmed that silica nanopaticles triggered DDR pathways leading to activate the G2/M cell cycle checkpoint. As shown in Figure 9, we found that Cdc25C, Cdc2 and cyclin B1 were remarkable suppressed in HUVECs after exposure to silica nanoparticles for 24 h, while Chk1 was significantly increased. Checkpoint kinase 1 (Chk1), which is an essential kinase required to preserve genome stability, is activated in response to DNA damage and is involved in the cell cycle checkpoint control, DNA damage repair and DNA damage-induced apoptosis [53], [54]. In particular, Chk1 is mainly responsible for the G2/M DNA damage checkpoint signal transduction pathway [55], [56]. Upon to DDR, Chk1 is activated and inhibits the activation of the downstream target of Cdc25C, resulted in the downregulation of cyclinB1/Cdc2 kinase [57]. Cdc2 and cyclin B1 are essential for the entry of cells into mitosis. Cdc2 is inactive as a monomer and must bind with cyclin B1 during the G2/M transition. Inhibition of cyclin B1/Cdc2 complex resulted in a directly G2/M arrest [58]. Thus, we could confirm that the mechanisms of silica nanoparticles induced endothelial cells toxic effect was through activating the Chk1-dependent G2/M DNA damage checkpoint signaling pathway. The molecular mechanism obtained from our study may add information to the epidemiologic data that exposure to ultrafine particles is a significant risk for the development of cardiovascular diseases.

## Conclusions

In summary, the present study demonstrates that silica nanoparticles induce ROS generation and DDR, caused endothelial cells toxic effect through Chk1-dependent G2/M DNA damage checkpoint signaling pathway. Thus, our findings suggest that exposure to silica nanoparticles could be a potential hazardous factor for the development of cardiovascular diseases, more studies of relation between silica nanoparticles exposure, adverse effects and biological mechanisms are needed for the safety evaluation and biomedical application of nanoparticles.

## References

[1] Kumar R, Roy I, Ohulchanskky TY, Vathy LA, Bergey EJ, et al (2010) In vivo biodistribution and clearance studies using multimodal organically modified silica nanoparticles. ACS Nano 4: 699–708. 20088598 10.1021/nn901146y PMC2827663

[2] Li Z, Barnes JC, Bosoy A, Stoddart JF, Zink JI (2012) Mesoporous silica nanoparticles in biomedical applications. Chem Soc Rev 41: 2590–605. 22216418 10.1039/c1cs15246g

[3] Barandeh F, Nguyen PL, Kumar R, Iacobucci GJ, Kuznicki ML, et al (2012) Organically modified silica nanoparticles are biocompatible and can be targeted to neurons in vivo. PLoS One 7: e29424. 22238611 10.1371/journal.pone.0029424 PMC3250438

[4] Lee JE, Lee N, Kim T, Kim J, Hyeon T (2011) Multifunctional mesoporous silica nanocomposite nanoparticles for theranostic applications. Acc Chem Res 44: 893–902. 21848274 10.1021/ar2000259

[5] Kang JH, Keller JJ, Chen CS, Lin HC (2012) Asian dust storm events are associated with an acute increase in pneumonia hospitalization. Ann Epidemiol 22: 257–263. 22391266 10.1016/j.annepidem.2012.02.008

[6] Zhao Y, Chen Z, Shen X, Zhang X (2011) Kinetics and mechanisms of heterogeneous reaction of gaseous hydrogen peroxide on mineral oxide particles. Environ Sci Technol 45: 3317–3324. 21428282 10.1021/es104107c

[7] Pope CA 3rd, Burnett RT, Thurston GD, Thun MJ, Calle EE, et al (2004) Cardiovascular mortality and long-term exposure to particulate air pollution: epidemiological evidence of general pathophysiological pathways of disease. Circulation 109: 71–7. 14676145 10.1161/01.CIR.0000108927.80044.7F

[8] Schneider A, Hampel R, Ibald-Mulli A, Zareba W, Schmidt G, et al (2010) Changes in deceleration capacity of heart rate and heart rate variability induced by ambient air pollution in individuals with coronary artery disease. Part Fibre Toxicol 7: 29. 20929559 10.1186/1743-8977-7-29 PMC2958976

[9] Brook RD, Rajagopalan S, Pope CA 3rd, Brook JR, Bhatnagar A, et al (2010) Particulate matter air pollution and cardiovascular disease: An update to the scientific statement from the American Heart Association. Circulation 121: 2331–2378. 20458016 10.1161/CIR.0b013e3181dbece1

[10] Mills NL, Tornqvist H, Gonzalez MC, Vink E, Robinson SD, et al (2007) Ischemic and thrombotic effects of dilute diesel-exhaust inhalation in men with coronary heart disease. N Engl J Med 357: 1075–82. 17855668 10.1056/NEJMoa066314

[11] Alom-Ruiz SP, Anilkumar N, Shah AM (2008) Reactive oxygen species and endothelial activation. Antioxid Redox Signal 10: 1089–1100. 18315494 10.1089/ars.2007.2007

[12] Kadam SS, Tiwari S, Bhonde RR (2009) Simultaneous isolation of vascular endothelial cells and mesenchymal stem cells from the human umbilical cord. In Vitro Cell Dev Biol Anim 45: 23–27. 19057971 10.1007/s11626-008-9155-4

[13] Badr G, Al-Sadoon MK, Abdel-Maksoud MA, Rabah DM, El-Toni AM (2012) Cellular and Molecular Mechanisms Underlie the Anti-Tumor Activities Exerted by Walterinnesia aegyptia Venom Combined with Silica Nanoparticles against Multiple Myeloma Cancer Cell Types. PLoS One 7: e51661. 23251606 10.1371/journal.pone.0051661 PMC3518476

[14] Sandberg WJ, Lag M, Holme JA, Friede B, Gualtieri M, et al (2012) Comparison of non-crystalline silica nanoparticles in IL-1beta release from macrophages. Part Fibre Toxicol 9: 32. 22882971 10.1186/1743-8977-9-32 PMC3441334

[15] Rabolli V, Thomassen LC, Princen C, Napierska D, Gonzalez L, et al (2010) Influence of size, surface area and microporosity on the in vitro cytotoxic activity of amorphous silica nanoparticles in different cell types. Nanotoxicology 4: 307–318. 20795912 10.3109/17435390.2010.482749

[16] Bauer AT, Strozyk EA, Gorzelanny C, Westerhausen C, Desch A, et al (2011) Cytotoxicity of silica nanoparticles through exocytosis of von Willebrand factor and necrotic cell death in primary human endothelial cells. Biomaterials 32: 8385–8393. 21840590 10.1016/j.biomaterials.2011.07.078

[17] Corbalan JJ, Medina C, Jacoby A, Malinski T, Radomski MW (2011) Amorphous silica nanoparticles trigger nitric oxide/peroxynitrite imbalance in human endothelial cells: inflammatory and cytotoxic effects. Int J Nanomedicine 6: 2821–2835. 22131828 10.2147/IJN.S25071 PMC3224709

[18] Liu X, Sun J (2010) Endothelial cells dysfunction induced by silica nanoparticles through oxidative stress via JNK/P53 and NF-kappaB pathways. Biomaterials 31: 8198–8209. 20727582 10.1016/j.biomaterials.2010.07.069

[19] Li Y, Sun L, Jin M, Du Z, Liu X, et al (2011) Size-dependent cytotoxicity of amorphous silica nanoparticles in human hepatoma HepG2 cells. Toxicol In Vitro 25: 1343–1352. 21575712 10.1016/j.tiv.2011.05.003

[20] Hoeijmakers JH (2001) Genome maintenance mechanisms for preventing cancer. Nature 411: 366–374. 11357144 10.1038/35077232

[21] Smith J, Tho LM, Xu N, Gillespie DA (2010) The ATM-Chk2 and ATR-Chk1 pathways in DNA damage signaling and cancer. Adv Cancer Res 108: 73–112. 21034966 10.1016/B978-0-12-380888-2.00003-0

[22] Lobrich M, Jeggo PA (2007) The impact of a negligent G2/M checkpoint on genomic instability and cancer induction. Nat Rev Cancer 7: 861–869. 17943134 10.1038/nrc2248

[23] Ciccia A, Elledge SJ (2010) The DNA damage response: making it safe to play with knives. Mol Cell 40: 179–204. 20965415 10.1016/j.molcel.2010.09.019 PMC2988877

[24] Liang Y, Lin SY, Brunicardi FC, Goss J, Li K (2009) DNA damage response pathways in tumor suppression and cancer treatment. World J Surg 33: 661–666. 19034564 10.1007/s00268-008-9840-1

[25] Sun L, Li Y, Liu X, Jin M, Zhang L, et al (2011) Cytotoxicity and mitochondrial damage caused by silica nanoparticles. Toxicol In Vitro 25: 1619–1629. 21723938 10.1016/j.tiv.2011.06.012

[26] Xu J, Sun L, Li J, Liang J, Zhang H, et al (2011) FITC and Ru(phen)_3_ ^2+^ co-doped silica particles as visualized ratiometric pH indicator. Nanoscale Res Lett 6: 561. 22027093 10.1186/1556-276X-6-561 PMC3220666

[27] Napierska D, Thomassen LC, Rabolli V, Lison D, Gonzalez L, et al (2009) Size-dependent cytotoxicity of monodisperse silica nanoparticles in human endothelial cells. Small 5: 846–853. 19288475 10.1002/smll.200800461

[28] Jiang J, Oberdörster G, Biswas P (2009) Characterization of size, surface charge, and agglomeration state of nanoparticle dispersions for toxicological studies. Journal of Nanoparticle Research 11: 77–89.

[29] Sharifi S, Behzadi S, Laurent S, Forrest ML, Stroeve P, et al (2012) Toxicity of nanomaterials. Chem Soc Rev 41: 2323–2343. 22170510 10.1039/c1cs15188f PMC4703119

[30] Smith AM, Duan H, Mohs AM, Nie S (2008) Bioconjugated quantum dots for in vivo molecular and cellular imaging. Adv Drug Deliv Rev 60: 1226–1240. 18495291 10.1016/j.addr.2008.03.015 PMC2649798

[31] Fotakis G, Timbrell JA (2006) In vitro cytotoxicity assays: comparison of LDH, neutral red, MTT and protein assay in hepatoma cell lines following exposure to cadmium chloride. Toxicol Lett 160: 171–177. 16111842 10.1016/j.toxlet.2005.07.001

[32] Inayat-Hussain SH, Chan KM, Rajab NF, Din LB, Chow SC, et al (2010) Goniothalamin-induced oxidative stress, DNA damage and apoptosis via caspase-2 independent and Bcl-2 independent pathways in Jurkat T-cells. Toxicol Lett 193: 108–114. 20026395 10.1016/j.toxlet.2009.12.010 PMC2828539

[33] Tedgui A, Mallat Z (2003) Apoptosis, a major determinant of atherothrombosis. Arch Mal Coeur Vaiss 96: 671–675. 12868350

[34] Nel A, Xia T, Madler L, Li N (2006) Toxic potential of materials at the nanolevel. Science 311: 622–627. 16456071 10.1126/science.1114397

[35] Napierska D, Thomassen LC, Lison D, Martens JA, Hoet PH (2010) The nanosilica hazard: another variable entity. Part Fibre Toxicol 7: 39. 21126379 10.1186/1743-8977-7-39 PMC3014868

[36] Shang Y, Zhu T, Li Y, Zhao J (2009) Size-dependent hydroxyl radicals generation induced by SiO 2 ultra-fine particles: The role of surface iron. Science in China Series B: Chemistry 52: 1033–1041.

[37] Valko M, Leibfritz D, Moncol J, Cronin MT, Mazur M, et al (2007) Free radicals and antioxidants in normal physiological functions and human disease. Int J Biochem Cell Biol 39: 44–84. 16978905 10.1016/j.biocel.2006.07.001

[38] Zhang Y, Hu L, Yu D, Gao C (2010) Influence of silica particle internalization on adhesion and migration of human dermal fibroblasts. Biomaterials 31: 8465–8474. 20701964 10.1016/j.biomaterials.2010.07.060

[39] Choi SJ, Oh JM, Choy JH (2009) Toxicological effects of inorganic nanoparticles on human lung cancer A549 cells. J Inorg Biochem 103: 463–471. 19181388 10.1016/j.jinorgbio.2008.12.017

[40] Eom HJ, Choi J (2009) Oxidative stress of silica nanoparticles in human bronchial epithelial cell, Beas-2B. Toxicol In Vitro 23: 1326–1332. 19602432 10.1016/j.tiv.2009.07.010

[41] Kowaltowski AJ, de Souza-Pinto NC, Castilho RF, Vercesi AE (2009) Mitochondria and reactive oxygen species. Free Radic Biol Med 47: 333–343. 19427899 10.1016/j.freeradbiomed.2009.05.004

[42] Kroemer G, Galluzzi L, Brenner C (2007) Mitochondrial membrane permeabilization in cell death. Physiol Rev 87: 99–163. 17237344 10.1152/physrev.00013.2006

[43] Jezek P, Hlavata L (2005) Mitochondria in homeostasis of reactive oxygen species in cell, tissues, and organism. Int J Biochem Cell Biol 37: 2478–2503. 16103002 10.1016/j.biocel.2005.05.013

[44] Montero AJ, Jassem J (2011) Cellular redox pathways as a therapeutic target in the treatment of cancer. Drugs 71: 1385–1396. 21812504 10.2165/11592590-000000000-00000

[45] Wan R, Mo Y, Feng L, Chien S, Tollerud DJ, et al (2012) DNA damage caused by metal nanoparticles: involvement of oxidative stress and activation of ATM. Chem Res Toxicol 25: 1402–1411. 22559321 10.1021/tx200513t PMC3398242

[46] Nabeshi H, Yoshikawa T, Matsuyama K, Nakazato Y, Tochigi S, et al (2011) Amorphous nanosilica induce endocytosis-dependent ROS generation and DNA damage in human keratinocytes. Part Fibre Toxicol 8: 1. 21235812 10.1186/1743-8977-8-1 PMC3030505

[47] Park MV, Verharen HW, Zwart E, Hernandez LG, van Benthem J, et al (2011) Genotoxicity evaluation of amorphous silica nanoparticles of different sizes using the micronucleus and the plasmid lacZ gene mutation assay. Nanotoxicology 5: 168–181. 20735203 10.3109/17435390.2010.506016

[48] Nabeshi H, Yoshikawa T, Matsuyama K, Nakazato Y, Matsuo K, et al (2011) Systemic distribution, nuclear entry and cytotoxicity of amorphous nanosilica following topical application. Biomaterials 32: 2713–2724. 21262533 10.1016/j.biomaterials.2010.12.042

[49] Wang J, Engle S, Zhang Y (2011) A new in vitro system for activating the cell cycle checkpoint. Cell Cycle 10: 500–506. 21252628 10.4161/cc.10.3.14753 PMC3050501

[50] Huang S, Chueh PJ, Lin YW, Shih TS, Chuang SM (2009) Disturbed mitotic progression and genome segregation are involved in cell transformation mediated by nano-TiO2 long-term exposure. Toxicol Appl Pharmacol 241: 182–194. 19695278 10.1016/j.taap.2009.08.013

[51] Schonn I, Hennesen J, Dartsch DC (2010) Cellular responses to etoposide: cell death despite cell cycle arrest and repair of DNA damage. Apoptosis 15: 162–172. 20041303 10.1007/s10495-009-0440-9

[52] Chen Y, Poon RY (2008) The multiple checkpoint functions of CHK1 and CHK2 in maintenance of genome stability. Front Biosci 13: 5016–5029. 18508566 10.2741/3060

[53] Reinhardt HC, Yaffe MB (2009) Kinases that control the cell cycle in response to DNA damage: Chk1, Chk2, and MK2. Curr Opin Cell Biol 21: 245–255. 19230643 10.1016/j.ceb.2009.01.018 PMC2699687

[54] Smits VA, Reaper PM, Jackson SP (2006) Rapid PIKK-dependent release of Chk1 from chromatin promotes the DNA-damage checkpoint response. Curr Biol 16: 150–159. 16360315 10.1016/j.cub.2005.11.066

[55] Syljuasen RG, Sorensen CS, Hansen LT, Fugger K, Lundin C, et al (2005) Inhibition of human Chk1 causes increased initiation of DNA replication, phosphorylation of ATR targets, and DNA breakage. Mol Cell Biol 25: 3553–3562. 15831461 10.1128/MCB.25.9.3553-3562.2005 PMC1084285

[56] Loffler H, Bochtler T, Fritz B, Tews B, Ho AD, et al (2007) DNA damage-induced accumulation of centrosomal Chk1 contributes to its checkpoint function. Cell Cycle 6: 2541–2548. 17726372 10.4161/cc.6.20.4810

[57] Lam MH, Rosen JM (2004) Chk1 versus Cdc25: chking one's levels of cellular proliferation. Cell Cycle 3: 1355–1357. 15483403 10.4161/cc.3.11.1225

[58] Malumbres M, Barbacid M (2005) Mammalian cyclin-dependent kinases. Trends Biochem Sci 30: 630–641. 16236519 10.1016/j.tibs.2005.09.005

